# Supplementary material for: Antibacterial Activity of a Promising Antibacterial Agent: 22-(4-(2-(4-Nitrophenyl-piperazin-1-yl)-acetyl)-piperazin-1-yl)-22-deoxypleuromutilin
Source: Molecules. 2021 Jun 8;26(12):3502. doi: 10.3390/molecules26123502 (PMC8227856; doi:10.3390/molecules26123502)
Supplement: Supplementary file 1 [file molecules-26-03502-s001.zip › molecules-1219364-supplementary.pdf]

Supplementary Materials

Antibacterial Activity of a Promising Antibacterial Agent: 22-(4-(2-(4-Nitrophenyl-piperazin-1-yl)-acetyl)-piperazin-1-yl)-22-deoxypleuromutilin

Xiang-Yi Zuo[1,†], Hong Gao[1,†], Mei-Ling Gao[1], Zhen Jin[1] and You-Zhi Tang[1,2,*]

[1] Guangdong Provincial Key Laboratory of Veterinary Pharmaceutics Development and Safety Evaluation, College of Veterinary Medicine, South China Agricultural University, Guangzhou 510642, China
[2] Guangdong Laboratory for Lingnan Modern Agriculture, Guangzhou, 510642, China
[†] These authors equally contributed to this paper.
[*] Correspondence: youzhitang@scau.edu.cn; Fax: +86 20 85280665.

Equipment and analysis conditions of pharmacokinetic studies

The HPLC system consisted of an Agilent Technologies HPLC system (California, USA) and an auto-sampler. Chromatographic separation was carried out on a Waters Symmetry C18 (3.5 μm, 2.1 × 50 mm) reversed-phase Column (Massachusetts, USA) maintained at 40 °C. The mobile phase was composed of solvent A (formic acid aqueous solution, 0.01%, *v*/*v*) and solvent B (acetonitrile), with a gradient elution of 0.0 min, 85:15; 0.5 min, 5:95; 3.0 min, 5:95; 3.5 min, 85:15; 9.0 min, 85:15. The flow rate was 0.25 mL/min and the sample injection volume was 5 μL. The samples were kept at 8 °C in the autosampler.

Mass spectrometric detection was carried out on an AB Sciex API 4000 triple quadrupole mass spectrometer equipped with an electrospray ionization (ESI) source (AB Sciex, Framingham, MA, USA). Positive spray ionization mode was used to detect NPDM. Analyst 1.6 software (Agilent Technologies, USA) was used to acquire and analyze the data. The optimum instrument parameters of NPDM were as follows: ion spray voltage, 4500 V; nebulizer gas, 55 psi; ion source gas 2, 40 L/min; curtain gas, 20 psi; turbo heater temperature, 600 °C. The quantification and characterization of NPDM were completed by multiple reaction monitoring (MRM), and the MRM parameters of NPDM were shown in supplemental data (see Table S1).

Table S1. MRM parameters of NPDM.

Parent ion (*m*/*z*)	Product ion (*m*/*z*)	DT (ms)	DP (V)	EP (V)	CE (V)	CXP (V)
694.5*	392.4*	100	140	10	31	12
694.5	285.3	100	130	10	34	12

Ion pairs with * are quantitative ion pairs. The excimer ion of the parent ion is $[M + H]^+$. DT: dwell time, DP: declustering potential, CE: collision energy, EP: entrance potential, CXP: collision cell exit potential.

Method validation of pharmacokinetic studies

The blank plasma matrix was prepared from mouse blank plasma by the method described in 2.8. and mixed with different concentrations of NPDM standard solution to obtain the final concentrations of 0~500 ng/mL. A calibration curve was drawn by plotting the peak area (y) of NPDM versus concentration (x) to determine the linearity of the method.

In order to determine the accuracy and precision of the method, three concentrations of NPDM standard solution (10, 100, and 500 ng/mL) were added to the matrix blank samples for extraction recovery, intra-day and inter-day analysis. The coefficient of variation was calculated according to Equation (S1).

$$\mathrm{CV}\ (\text{or } RSD) = \frac{\sqrt{\sum_{i=1}^{n}(Xi-\bar{X})^2/(n-1)}}{\bar{X}} \times 100\% \tag{S1}$$

X_i: measured compound concentration of each sample; $\bar{X}$: the average of several parallel samples at the same concentration; n: the number of parallel samples.

The limit of detection (LOD) and the limit of quantification (LOQ) were calculated by performing a serial dilution of standard solution spiked in matrix-blank samples.

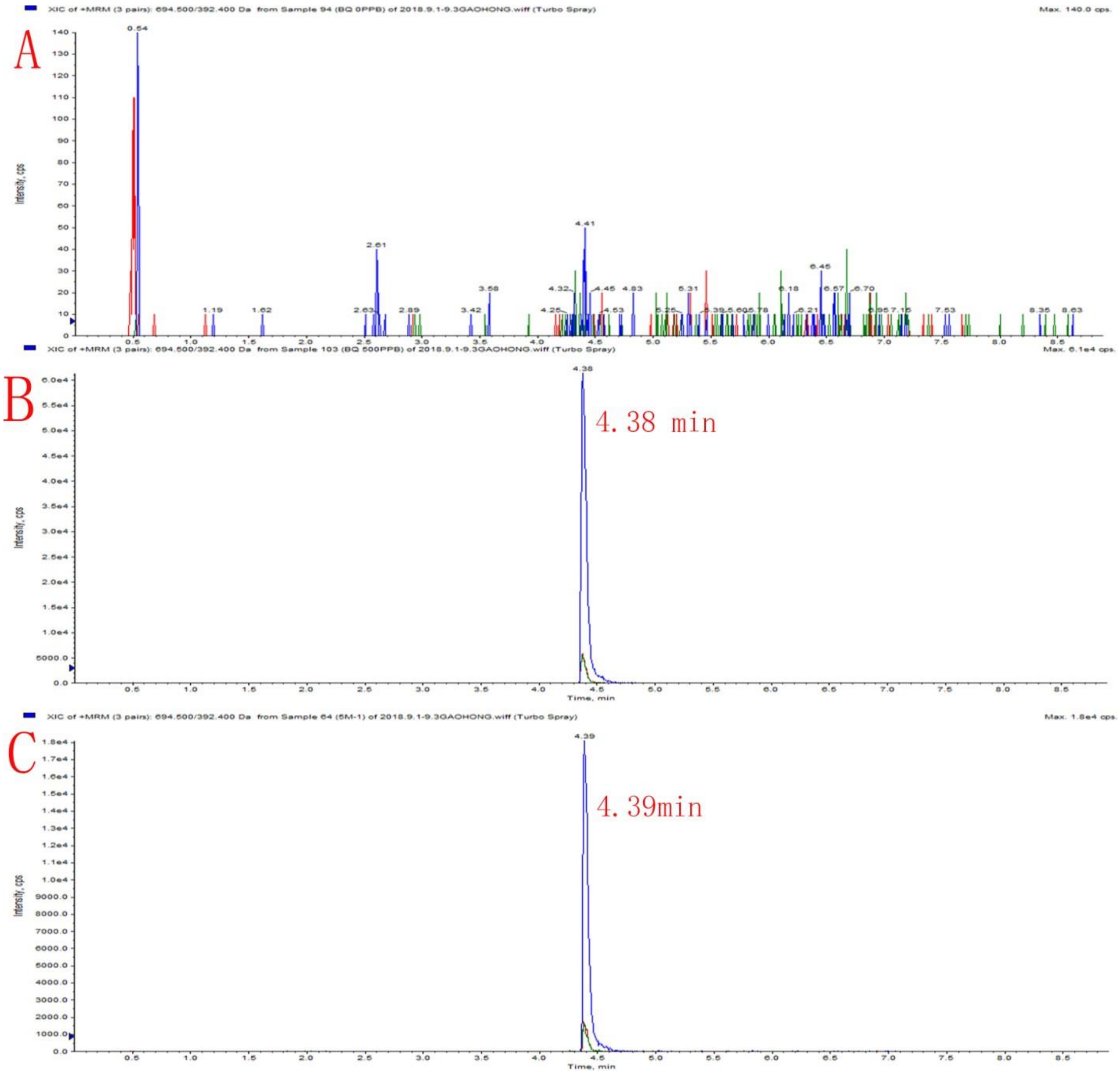

Figure S1. LC-MS/MS chromatogram of a blank plasma sample (**A**), a blank plasma sample added 500 ng/mL NPDM (**B**), and a plasma sample 5 min after administration with NPDM (**C**).

Table S1. Intra- and inter- day assay precision and accuracy for NPDM.

Concentration (ng/mL)	intra-day		inter-day	
	Mean ± S.D (*n* = 5)	C.V (%)	Mean ± SD (n = 3)	C.V (%)
	8.49 ± 0.68	8.04		
10	8.67 ± 0.76	8.74	9.26 ± 1.18	12.77
	10.62 ± 0.52	4.87		
	82.60 ± 3.58	4.33		
100	87.40 ± 2.40	2.75	89.67 ± 8.76	9.75
	99.60 ± 5.28	5.30		
	417.00 ± 9.08	2.19		
500	430.80 ± 17.20	3.99	447.20 ± 40.94	9.16
	493.80 ± 16.95	3.43		
